# Spatially-Localized Functionalization on Nanostructured Surfaces for Enhanced Plasmonic Sensing Efficacy

**DOI:** 10.3390/nano12203586

**Published:** 2022-10-13

**Authors:** Jean-François Bryche, Marlo Vega, Agnès Tempez, Thibault Brulé, Thomas Carlier, Julien Moreau, Marc Chaigneau, Paul G. Charette, Michael Canva

**Affiliations:** 1 Laboratoire Nanotechnologies Nanosystèmes (LN2-IRL 3463)-CNRS, Université de Sherbrooke, 3000 Boulevard de l’Université, Sherbrooke, QC J1K OA5, Canada; 2 Institut Interdisciplinaire d’Innovation Technologique (3IT), 3000 Boulevard de l’Université, Sherbrooke, QC J1K OA5, Canada; 3 Laboratoire Charles Fabry—Institut d’Optique Graduate School, Université Paris-Saclay, CNRS, 91120 Palaiseau, France; 4 Horiba FRANCE SAS, 91120 Palaiseau, France

**Keywords:** Raman spectroscopy, SERS, TERS, plasmonics, nanostructures, surface functionalization

## Abstract

This work demonstrates the enhancement in plasmonic sensing efficacy resulting from spatially-localized functionalization on nanostructured surfaces, whereby probe molecules are concentrated in areas of high field concentration. Comparison between SERS measurements on nanostructured surfaces (arrays of nanodisks 110 and 220 nm in diameter) with homogeneous and spatially-localized functionalization with thiophenol demonstrates that the Raman signal originates mainly from areas with high field concentration. TERS measurements with 10 nm spatial resolution confirm the field distribution profiles predicted by the numerical modeling. Though this enhancement in plasmonic sensing efficacy is demonstrated with SERS, results apply equally well to any type of optical/plasmonic sensing on functionalized surfaces with nanostructuring.

## 1. Introduction

Improving photonic biosensor performance remains an ongoing challenge, requiring a comprehensive understanding of the physical principles involved and accurate characterization methods. Enabled by plasmonic nanostructures, surface-enhanced Raman spectroscopy (SERS) has led to significant amplification of the Raman signal [1,2,3,4]. Indeed, plasmonic nanostructures can confine electromagnetic fields beyond the diffraction limit and have enabled SERS to reach single-molecule detection [5]. Optimization on the nanostructure geometry can lead to a high electric field [6,7]. In particular, gold nanodisk arrays fabricated atop continuous gold films on glass substrates have recently shown promising results in SERS [2,8,9] as well as in SPR [9,10] due to the formation of hybridized plasmonic modes. The continuous metal layer sustains propagating surface plasmons while the nanostructures sustain localized surface plasmon resonances. Coupling between them occurs under specific conditions and gives rise to hybrid modes with improved characteristics for sensing [11,12]. Other important phenomena may also warrant consideration regarding their influence on the plasmonic response such as band structure, permittivity, size, and localized particle/lattice rearrangements [13,14,15,16].

Numerical modeling of nanostructured substrates predicts that the Raman signal is most intense from molecules located at the edges of the nanostructures due to the field concentration. This suggests that deliberate and accurate positioning of probe molecules at locations of high field concentration on the nanostructures could be a way to optimize detection sensitivity [17] and response time, especially at low molecular concentrations. In addition, the design of our sample composed of gold nanostructures on gold film is compatible with SPR biosensors and could overcome the limit of detection with accurate positioning of the molecule under conditions of very low target concentrations. Indeed, selective functionalization can exclude the disturbance from SERS-inactive molecules outside the hot spot.

To experimentally confirm model predictions and quantify the SERS sensitivity enhancement resulting from spatially resolved positioning of probe molecules at the nanoscale, a very high spatial resolution measurement tool is required.

Tip-enhanced Raman spectroscopy (TERS) has been used to study and map the electromagnetic near-field surrounding metal nanostructures [18] and multipolar plasmonic resonance modes from nanoparticles [19]. Recently, several factors that had limited spatial resolution in TERS have been addressed from both experimental and theoretical points of view [20]. Based on the tip selection, experimental configuration, and grafting of a molecule on the tip, TERS resolution can now achieve spatial resolutions from tens of nanometers down to a few angstroms [21,22,23,24]. As a result, TERS has been applied in a variety of demanding applications of surface characterization, catalysis, and single-molecule detection [25]. TERS instrument configurations and applications have recently been reviewed [26,27]. Field density and spatial distribution in the local neighborhood of nanoscale surface geometry features such as edges or surface heterogeneities have been precisely mapped with TERS [24,28,29,30], with studies focusing on the impact of tip size [31], commercial vs. custom-made tips [32], and positioning [33,34,35]. TERS is therefore ideally suited to characterize nanostructured SERS substrates to determine the optimal positioning of probe molecules on the sensor surface.

**Figure 1:**
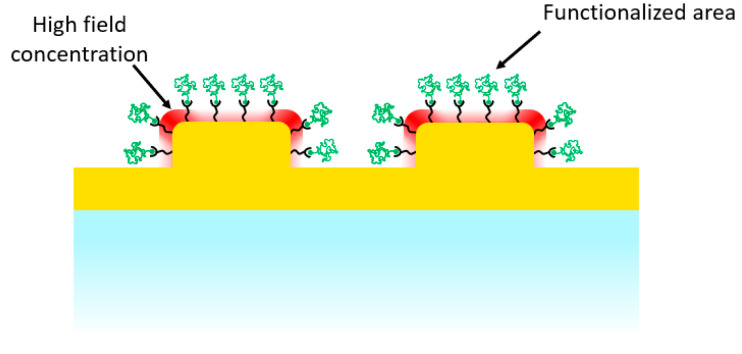
Schematic diagram of selectively positioned probe molecules near areas of high field concentration surrounding gold nanostructures atop a continuous metal film.

In this work, we demonstrate the impact on the SERS response of selectively positioning probe molecules near areas of high field concentration surrounding gold nanostructures atop a continuous metal film, as shown in Figure 1. Numerical simulations were performed to determine the field profile surrounding the nanostructures. TERS measurements of the Raman signal with sub-10 nm resolution confirmed the modeled field spatial distribution. Finally, measurements of the SERS response were conducted on samples with different spatially-localized surface functionalization with thiophenol, to demonstrate that molecules grafted to the surface in between nanostructures do not contribute significantly to the optical signal.

## 2. Materials and Methods

### 2.1. Numerical Modeling of Nanodisks Arrays

**Figure 2:**
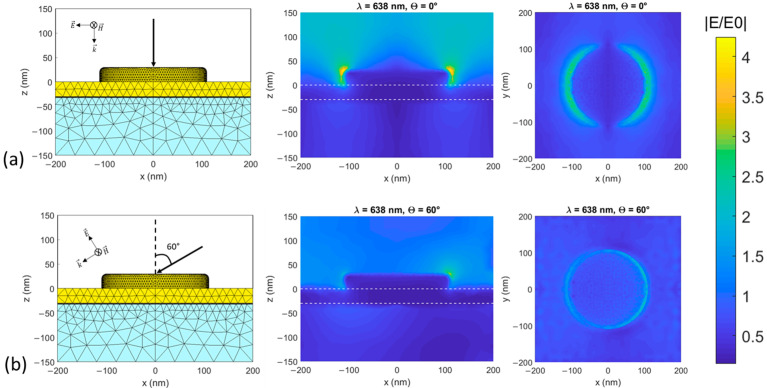
Numerical modeling results for λ = 638 nm. Left: domain meshing. Middle and right: Normalized electric field amplitude distribution for side (x, z) and top (x, y) views of the gold nanodisks (30 nm height, 220 nm diameter, 400 nm period, 10 nm radius edge rounding) in an infinite square array (400 nm period) atop a continuous gold film (30 nm height, 2 nm thickness titanium adhesion layer) on a glass substrate. The structure is excited with TM polarized light (p-polarized excitation light referenced to the surface plane (gold surface)), E0 is the incident electric field amplitude at 638 nm at: (**a**) 0° incidence and (**b**) 60° incidence. The horizontal dashed lines in the field profile side views (middle) show the top and bottom surfaces of the continuous gold film.

Based on previous work in our group, we conducted numerical simulations of electromagnetic field distribution at the surface of the bare gold nanostructures using an in-house developed numerical tool [36] that combines FEM (finite element method) with FMM (Fourier modal method). Simulations were conducted without the tip and molecules. Figure 2 shows the domain meshing (left) and normalized electric field amplitude distributions (middle: Side view, right: Top view) for an infinite gold disk array atop a continuous gold film on a glass substrate, excited at 638 nm at two incidence angles (normal and 60°). The geometry matches the experimental conditions described in the next section for the disk array (30 nm height, 220 nm diameter, 400 nm period, 10 nm radius edge rounding) and underlying metal film (30 nm height, 2 nm thickness titanium adhesion layer). Modeling results are shown for off-resonance conditions (the experimental nanostructured array resonance wavelength is 664 nm [2]) to reflect typical conditions of use, where the excitation wavelength is determined by the reporter molecule Raman characteristics. For both incidence angles, the field strength is greater at the edges of the nanodisks by up to a factor of 4 compared to areas between the disks.

### 2.2. Fabrication of Nanodisks Arrays

**Figure 3:**
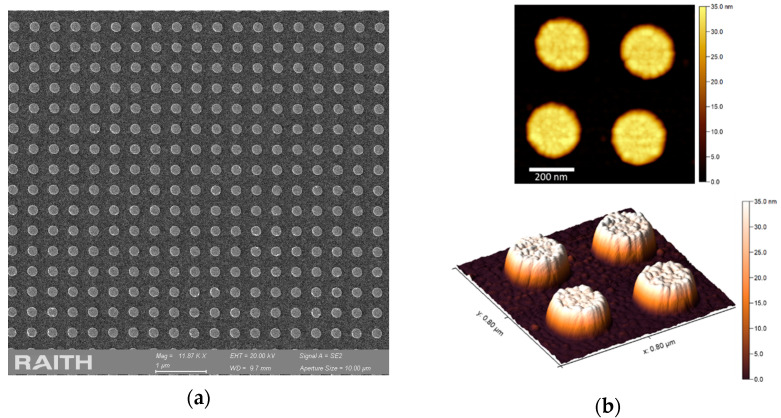
(**a**) SEM image of a gold nanodisk array (diameter = 220 nm, period = 400 nm) on a continuous gold film; (**b**) AFM measurements in 2D and 3D of the nanodisks.

We fabricated gold nanodisk arrays on gold films for two disk diameters: D = 110 nm and D = 220 nm. The nanostructures were prepared by e-beam lithography with a protocol published previously [2]. The SEM image in Figure 3a shows a well-defined array with D = 220 nm and a period (P) of 400 nm (see supplementary Figure S1 for 110 nm disks). AFM measurements (Figure 3b) of nanostructure disk height were 32 ± 5 nm, confirming the shape homogeneity and low surface roughness [28]. The underlying 30 nm gold film thickness was confirmed by ellipsometry (data not shown).

### 2.3. Surface Functionalization with Thiophenol

Samples were functionalized with a 0.1 mM solution of thiophenol (C_6_H_6_S) over a 2.5 h incubation time to saturate the gold surface. Samples were then rinsed with ethanol for 5 min and dried with nitrogen. We chose to use thiophenol molecules due to the large Raman cross-section of the benzene ring that facilitates its detection in SERS and TERS experiment. Thiophenol molecules have characteristic Raman peaks at 419/1000 cm^−1^ (out-of-plane C-C-C stretching), 1025 cm^−1^ (out-of-plane C-H stretching), 1075 cm^−1^ (C-C-C stretching in-plane and C-S stretching), and 1575 cm^−1^ (C-C stretching) [37,38]. As described further (in Section 3.2), samples were functionalized both with uniform surface coverage for TERS measurements and with spatially-localized coverage to validate the hypothesis of SERS molecular response improvement through nanostructuring.

### 2.4. TERS and SERS Measurement Methodology

**Figure 4:**
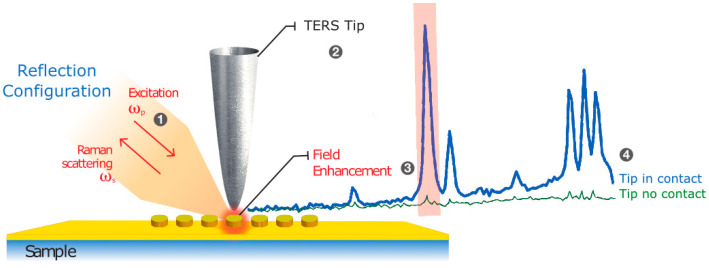
Schematic diagram of TERS measurement setup with laser excitation (1), tip (2), and Raman spectrum enhancement due to the local field around the tip (3,4).

TERS mapping of samples with homogeneous surface functionalization was used as a reporter mechanism to map the field distribution on the surface of the nanostructured samples and confirm the locations of high field concentration predicted by the numerical model. The TERS system consisted of an XploRA Nano system (HORIBA Scientific, Palaiseau, France) combining a scanning probe microscope (OmegaScope) with a Raman micro-spectrometer. Figure 4 shows a schematic illustration of the TERS measurement on a nanostructured sample. Laser excitation (638 nm, 80 µW, TM-polarized) was incident on the sample at an angle of 60° with respect to the surface normal. The laser light was focused using a 100× objective (NA = 0.7) mounted on a piezo scanner for positioning and focusing on the apex of the probe tip. The TERS probes were cantilever-based gold-coated AFM-TERS tips (OMNI TERS-SNC-Au, App Nano for HORIBA Scientific).

**Figure 5:**
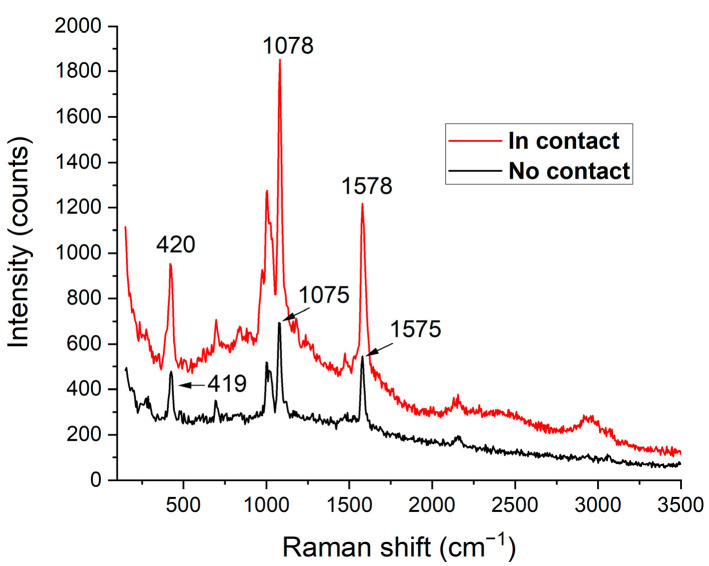
TERS Raman signal amplitude increase from a thiophenol-functionalized sample due to the tip in contact with the surface (TERS) at the center of a disk (“In contact”, red curve) versus without the tip (“No contact”, black curve). Laser excitation: 638 nm, 80 µW; Acquisition time: 5 s.

Lateral spatial resolution in TERS is defined as the full-width-at-half-maximum of the local field distribution around the tip in the plane of the surface: FWHM ≈ 0.87√Rd [33], where *R* is the tip radius and *d* is the height of the tip-to-surface gap. At *R* = 30 nm and *d* = 2 nm, FWHM resolution = 6.7 nm. As a compromise between image acquisition time, field uniformity, and spatial resolution, a scanning step size of 10 nm was chosen, hence the spatial resolution in the TERS images is 10 nm/pixel in both axes. TERS spectra were acquired with the tip in contact with the sample surface (typical interaction force of 2–10 nN, labeled “In contact” in Figure 4 and Figure 5). Between pixel measurements, the samples were displaced laterally in semi-contact mode to preserve tip sharpness. The TERS spectra were compared to spectra acquired with the tip retracted (no TERS local field enhancement, i.e., SERS, labeled “No contact” in Figure 4 and Figure 5). In both cases, acquisition times were 5 s per spectrum.

Finally, to confirm the Raman signal enhancement resulting from the combination of surface nanostructuring and spatially-localized surface functionalization, samples were characterized by SERS with an XploRA Raman micro-spectrometer using a 100× objective at 633 nm, 660 nm, and 785 nm as in previous work [2,8]. As shown in the Supplementary Materials (Figure S6), results were confirmed with a LabRAM Soleil Raman microscopy platform (HORIBA Scientific, Palaiseau, France). Acquisitions were carried out at 532 nm (9 mW), 638 nm (6 mW), and 785 nm (4 mW) excitation for 30 s over area of 144 μm^2^.

## 3. Results and Discussion

### 3.1. TERS and SERS Measurements on Samples with Homogeneous Surface Functionalization

Figure 5 shows the example spectra acquired from a point in the center of a nanodisk at an excitation wavelength of 638 nm on a thiophenol-functionalized sample: TERS spectrum (probe tip in contact with the surface, red curve) and spectrum due to the surface nanostructuring alone (tip withdrawn and no longer in contact with the surface, black curve, i.e., SERS). As expected, the TERS spectrum shows an increase in the Raman response.

This increase can be quantified for individual peaks in terms of contrast ratio, that is, the ratio *I_Tip-in_*/*I_Tip-out_*, where $I_{Tip-in}$ and $I_{Tip-out}$ are the Raman peak signal intensities with the tip in contact and withdrawn from the surface, respectively. After subtracting the background signal (smoothed baseline spectrum without the peaks, obtained by non-linear filtering) from both spectra, the peaks at 419, 1075, and 1575 cm^−1^ showed contrast ratios of 2.2, 2.8, and 2.5, respectively. Note that the $I_{Tip-out}$ measurements were consistent with previous SERS results obtained by our group on similar nanodisk arrays, at a slightly different excitation wavelength (633 nm) [2]. A slight widening and shift of the resonance peaks observed in the TERS spectrum is due to the perturbation of the Raman modes by the presence of the tip [31]. When the tip is in contact with the surface (TERS), the Raman signal in fact results from an excitation field having two components: A “far field” (FF) component from the laser source focused by the objective and an additive “near field” (NF) component from the tip, with respective spectral amplitude peaks *I_FF_* and *I_NF_*. Therefore, $I_{Tip-in}=I_{NF}+I_{FF}$ and $I_{Tip-out}=I_{FF}$.

The TERS signal increase can be normalized with respect to volume via the *TERS enhancement factor* (TERS_EF_), defined for a given peak as [39,40]:

$$TERS_{EF}=\left(\frac{I_{Tip-in}}{I_{Tip-out}}-1\right)\frac{V_{FF}}{V_{NF}}$$

where $V_{FF}$ and $V_{NF}$ are to the far-field and near-field excitation volumes, respectively. For the far field component, $V_{FF}=R_{focus}^{2}\pi b_{FF}$, with $R_{focus}$ and $b_{FF}$ the microscope objective focal spot radius and effective depth of focus. For the near field, $V_{NF}$ = $\left(R_{TERS}\right)2\pi b_{NF}$, with $R_{TERS}$ and $b_{NF}$ the radius of the TERS volume [40] and effective height of the near field, respectively. For laser illumination at angle α (60° in the experiments), the focal volume becomes elliptic, modifying the field distribution acting on the tip—this effect is accounted for by the cos(α) term below. For a thin layer of adsorbed molecules on the surface, $b_{FF}\approx b_{NF}$, therefore $\frac{V_{FF}}{V_{NF}}$ reduces to $R_{focus}^{2}/R_{TERS}^{2}$. Using the approximation $R_{TERS}=$ $\frac{1}{2}R_{tip}$ [41] with $R_{tip}\;$ the tip radius, the TERS enhancement factor reduces to [40]:

$$TERS_{EF}=\left(\frac{I_{NF}}{I_{FF}}\right){\left(\frac{R_{focus}}{\frac{1}{2}R_{tip}}\right)}^{2}\cos\left(\alpha \right)$$

In our experiments, $R_{focus}=1200$ nm, $R_{tip}=30$ nm, α=60°, yielding $TERS_{EF}$ values of 7 × 10^3^, 8.9 × 10^3^, and 8 × 10^3^ for the three peaks (419, 1075, and 1575 cm^−1^) for a 638 nm excitation. These values are in the range obtained by others with AFM-based TERS ($TERS_{EF}\leq 10^{4}$) [40]. Note that for tip diameters below 20 nm, $I_{Tip-in}=I_{NF}+2I_{FF}$, because the far-field contribution includes a mirror effect from the tip [39], which does not apply in our case.

Surface nanostructuring causes $TERS_{EF}$ values to be underestimated due to the SERS effect that enhances the $I_{Tip-out}$ “reference” (normally un-enhanced Raman response from a flat metal film). Indeed, measurements on unstructured gold films do not show characteristic peaks when the tip is withdrawn. Using the background signal as a reference in the denominator, $TERS_{EF}$ values are on the order of 3.9 × 10^6^, in agreement with results found by others [25,33]. Furthermore, previous SERS measurements realized on these nanodisk arrays by our group [2] yielded enhancement factor of 2.4 × 10^6^ corresponding to the far-field contribution (excitation wavelength of 633 nm instead of 638 nm). We confirm the $\frac{I_{NF}}{I_{FF}}$ ratio of 2.2 reported above by our indirect findings.

**Figure 6:**
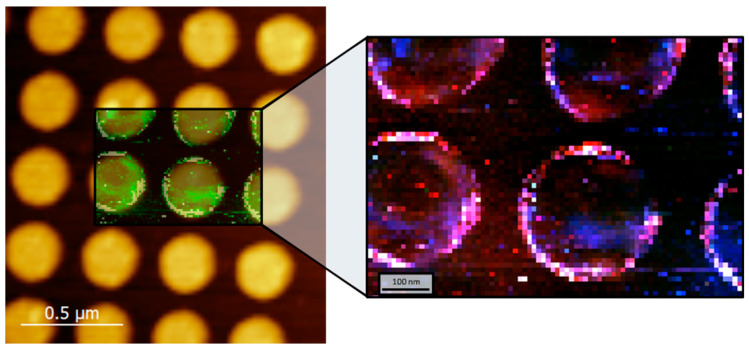
AFM image of a 220 nm diameter gold nanodisk array with TERS image overlay (638 nm excitation). The highest intensity TERS signals are located on the edges of the nanodisks. The colors in the TERS map are a superposition of the Raman signal contributions from three spectral bands of interest (see Supplementary Figure S2). The TERS image pixel size is 10 nm in both axes.

Figure 6 shows an AFM image (1650 × 1560 nm^2^) of a portion of a 220 nm diameter gold nanodisk array excited at 638 nm, with a composite TERS signal amplitude map overlay for the central area. The colors in the TERS map are a superposition of the Raman signal contributions from three spectral bands of interest (see Supplementary Figure S2). The highest intensity TERS signals are located on the edges of the nanodisks, as expected. These results agree with a recent lower spatial resolution study by others for a gold nanodisk array on a Si substrate functionalized with a MoS_2_ monolayer [34].

**Figure 7:**
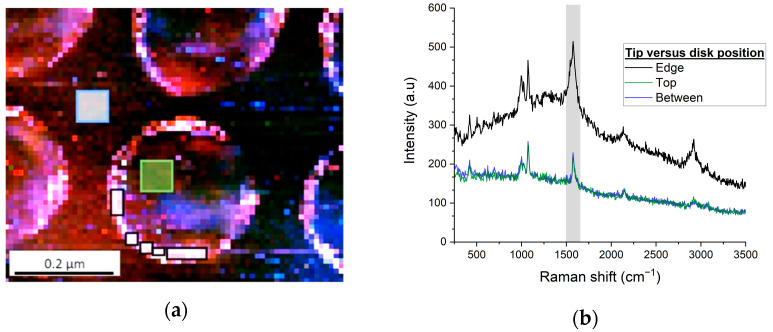
(**a**) TERS mapping of 220 nm gold nanodisk array excited at 638 nm (same image as Figure 6 with higher brightness); (**b**) Raman spectra averaged over 36 pixels for three distinct zones of surface topology: top-edge-between; note that signals from the top and between are almost indistinguishable. The outlines in (**a**) show the averaging areas (the area for the disk edge spectrum averaging is split into multiple sub-areas to conform to the curved shape).

TERS spectra from three distinct areas of the sample topology were compared: disk top surface, disk edge, and between disks, as shown in Figure 7. The spectra were averaged over ROIs of 36 pixels each (each pixel is a distinct TERS spectrum) as shown by the outlines in the figure (note that the ROI for the disk edge spectrum is split into multiple sub-areas to conform to the curved shape). As expected, the black curve corresponding to the nanostructure edges shows higher peak values and signal intensity overall versus the blue (top of disk) and green (between disks) curves (the latter two spectra are similar since they correspond to TERS gap-modes, i.e., tip interaction with a locally-flat surface [39]).

The ratio between Raman signal intensities at the edges versus the center of the nanostructures is defined as the signal *gain*, *G*, that is, the gain in biosensor sensitivity expected from the spatially-localized positioning of probe molecules. For the samples with 220 nm diameter disks, *G* = 2.1 ± 0.4 for the 1575 cm^−1^ peak. Note that for gain estimation at the edges, signal integration may average high and low intensities on either side of edges, resulting in a slight underestimation of the gain. Note also that gain values will depend on metal grain boundaries, tip thickness, excitation wavelength, and nanostructure geometry. Equivalent experiments performed on smaller nanodisks (D = 110 nm, see Figure S3) yielded *G* = 1.8 ± 0.3. These values agree with similar experiments by others on 100 nm gold nanodisks (height = 20 nm, period = 150 nm) where a gain *G* of 4.2 was observed [33].

The TERS measurements confirmed the correlation between areas of highest concentration of electric field at the edges of the nanostructures with highest Raman response. These results therefore strongly suggest that selective localization of probe molecules on the nanostructured sensor surface could indeed improve SERS sensing performance, especially at low target concentrations.

### 3.2. SERS Measurements on Samples with Spatially-Localized Surface Functionalization 

**Figure 8:**
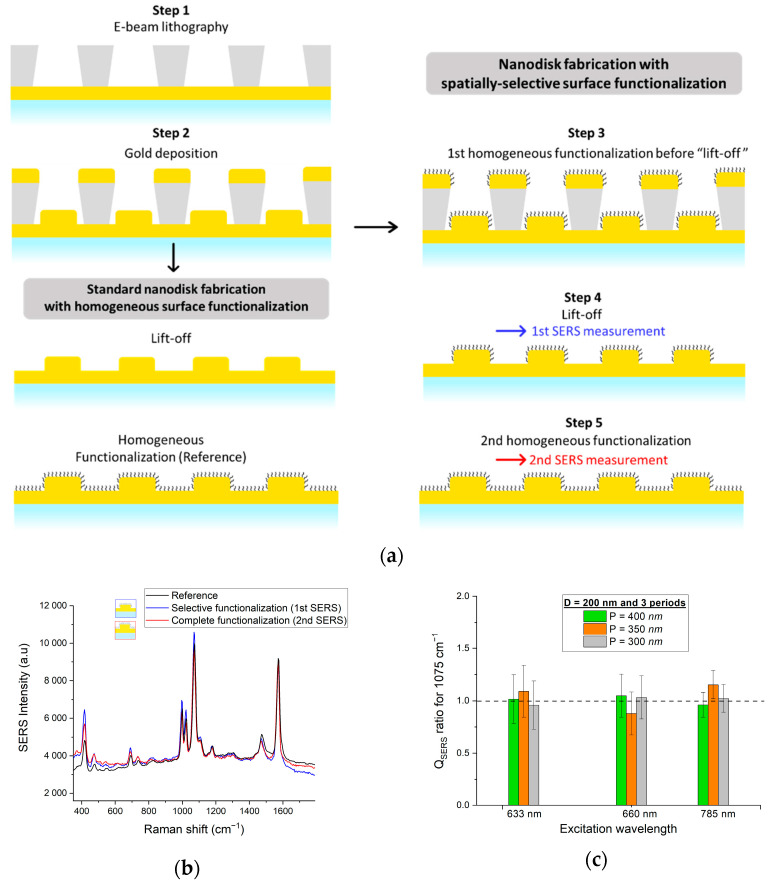
(**a**) Schematic representation of spatially-localized nanostructure functionalization based on a lift-off process; (**b**) superposition of reference, spatially-localized (step 4) and homogeneous (step 5) functionalization SERS spectra (an offset on the *y*-axis was applied to overlay the spectra); (**c**) ratio of SERS intensity (*Q_SERS_*) obtained for spatially-localized and homogeneous functionalization at three excitation wavelengths (633, 660, and 785 nm) and three disk array periods (300 nm, 350 nm, 400 nm) for the 1075 cm^−1^ peak.

As described in Figure 8a, we developed a method for spatially-localized surface functionalization that simply adds a simple step to our nanodisk array fabrication process [2]. The method greatly simplifies spatially-localized surface functionalization compared to previous work by others [42,43,44,45], though the surface chemistry must be compatible with immersion in acetone. Following e-beam lithography (step 1) and gold deposition (step 2), thiophenol was deposited homogeneously on the sample surface by incubation as described above (step 3). The resist (grey material in the figure) was then removed by lift-off in acetone (step 4), leaving only the disk top-edge surfaces with thiophenol functionalization. TERS measurements between the disks 24 h after step 4 confirmed that there was no measurable migration of thiophenol to the areas between the disks by surface diffusion so that the surface functionalization was stable over time (Supplementary Figure S4). As shown in step 5, homogeneously functionalized reference samples were obtained by post-processing with a second incubation in thiophenol. In the case of reference samples (on the left of Figure 8a), the lift-off process precedes the surface functionalization, resulting in homogeneous surface coverage.

The ratio of Raman signal intensities measured at steps 4 and 5 for the 1075 cm^−1^ peak, *Q_SERS_*, was used to assess the effectiveness of the spatially-localized functionalization on the SERS response, *Q_SERS_ =* $\frac{I_{step4}}{I_{step5}}$ (example spectrum shown in Figure 8b). A ratio of *Q_SERS_* = 1 implies that the second surface functionalization at step 5 did not add any significant signal contribution, and hence, that most of the Raman signal came from the selectively functionalized disk edges and top surfaces. A ratio of *Q_SERS_* < 1 implies that the functionalization between the disks added non-negligible Raman signal contributions. *Q_SERS_* values were calculated for three excitation wavelengths (633, 660, and 785 nm) for 220 nm diameter disk arrays with three different periods (300 nm, 350 nm, 400 nm). As shown in Figure 8c, a ratio of *Q_SERS_* = ~1 was obtained in all cases (the error bars correspond to an average of 10 spectra for each combination of wavelength and period obtained from distinct regions of the disk array encompassing four disks on average, typical standard deviation of ~10%). The experiment was also performed on arrays of 110 nm diameter disks and yielded similar results (see Figure S5 in the Supplementary Materials).

The sensor “capture efficacy” afforded by functionalization selectivity can be quantified by the fill factor, ρ (functionalized proportion of total surface area), defined as the ratio between the disk top surface area and the square of the array periodicity: ρ = (πD^2^)/(4P^2^). Disk vertical walls were neglected because of the high form factor (height ≪ diameter). For 220 nm diameter disks with a 400 nm periodicity, the fill factor was 23.8%, corresponding to gain of 4.2 (1/ρ) in capture efficacy. As such, under conditions of very low target concentration, i.e., where the bulk concentration of target molecules is insufficient to saturate the sensor surface, the SERS response of a selectively-functionalized nanostructured sensor could be up to 4.2× greater compared to a homogeneously functionalized device. Note that 110 nm disks with a 400 nm periodicity yielded an even lower fill factor (5.9%). However, compared to the 220 nm disk arrays, such small dimensional disk arrays are difficult to fabricate and would not be as well suited in practice to mass production. In addition to the increased sensor capture efficacy via spatially-selective surface functionalization, the SERS response under conditions of low target concentration could be enhanced further with tapered nanostructures [46] that produce stronger local fields compared to rounded structures, such as bowties [47,48], suspended bowties [49], or dimers with nanogaps [50]. However, there are still significant fabrication challenges involved in producing such structures uniformly over large surfaces at scale.

## 4. Conclusions

This work demonstrates the enhancement in SERS sensing efficacy resulting from spatially-localized functionalization, whereby probe molecules are concentrated in areas of high field concentration on the nanostructured sensor surface. As a result, under conditions of low target concentration, the SERS response will be enhanced compared to a homogeneously functionalized device. TERS measurements with 10 nm spatial resolution confirmed the field distribution profiles atop the nanostructured surface predicted by the numerical modeling. Comparison between SERS measurements from surfaces with homogeneous and spatially-localized functionalization confirmed that the Raman signal originates mainly from the areas with high field concentration (nanodisk edges). A simple method for spatially-localized functionalization involving a single additional step in a standard nanofabrication process was demonstrated, though the surface chemistry must be compatible with an acetone-based lift-off process. Though the enhancement in sensing efficacy was demonstrated with SERS, these results apply equally well to any type of optical/plasmonic sensing on functionalized nanostructured surfaces.

## Data Availability

The datasets generated during and/or analysed during the current study are available from the corresponding author on reasonable request.

## Supplementary Materials

The following supporting information can be downloaded at: https://www.mdpi.com/article/10.3390/nano12203586/s1. Additional SERS and TERS measurement results on 220 nm diameter disk arrays, as well as for 110 nm diameter disk arrays (AFM measurements, TERS characterization). Figure S1: AFM pictures in 2D and 3D views of the gold nanodisks on gold film for D = 110 nm and P = 400 nm.//Figure S2: TERS mapping of 220 nm gold nanodisks with 638 nm laser. Each region of the Raman spectra can be asso-ciated with area on the sample (a-b). Then, we can superpose the area (c). In the TERS image, the pixel size is 10 × 10 nm^2^//Figure S3. TERS mapping of 110 nm gold nanodisks with 638 nm laser. (b) Raman signal for three identified sample regions: Top – Edge – Between.//Figure S4. TERS intensities signal obtained from the top (red curve) and between (green curve) the nanostructures (220 nm gold nanodisks).//Figure S5. Ratio of SERS intensities between the first and second functionalization for three excitation wavelengths (633, 660, and 785 nm) for 1075 cm^−1^ characteristic peaks.//Figure S6. SERS spectra for two sizes of nanodisks (D = 110 and 220 nm) with a period of 400 nm. Three excitation wavelengths were used at 532, 638, and 785 nm.
