# Supplementary material for: Spatially-Localized Functionalization on Nanostructured Surfaces for Enhanced Plasmonic Sensing Efficacy
Source: Nanomaterials (Basel). 2022 Oct 13;12(20):3586. doi: 10.3390/nano12203586 (PMC9609756; doi:10.3390/nano12203586)
Supplement: Supplementary file 1 [file nanomaterials-12-03586-s001.zip › nanomaterials-1928477-supplementary.pdf]

nanomaterials

MDPI

Supplementary materials

Spatially-Localized Functionalization on Nanostructured Surfaces for Enhanced Plasmonic Sensing Efficacy

Jean-François Bryche [1,2,*], Marlo Vega [1,2,3], Agnès Tempez [4], Thibault Brulé [4], Thomas Carlier [4], Julien Moreau [3], Marc Chaigneau [4], Paul G. Charette [1,2] and Michael Canva [1,2]

1 Laboratoire Nanotechnologies Nanosystèmes (LN2-IRL 3463)-CNRS, Université de Sherbrooke, 3000 Boulevard de l'université, Sherbrooke, QC J1K OA5, Canada
2 Institut Interdisciplinaire d'Innovation Technologique (3IT), 3000 Boulevard de l'université, Sherbrooke, QC J1K OA5, Canada
3 Laboratoire Charles Fabry—Institut d'Optique Graduate School, Université Paris-Saclay, CNRS, Palaiseau, 91120, France
4 Horiba FRANCE SAS, Palaiseau, 91120, France

* Correspondence: jean-francois.bryche@usherbrooke.ca

1.1. AFM measurement

As presented in the main article, a similar analysis has been conducted with smaller gold nanodisks (D=110 nm diameter and P=400 nm). The AFM pictures are shown in 2D and 3D views in figure S1. The structures are well defined, apart from a slight rugosity at the top surface.

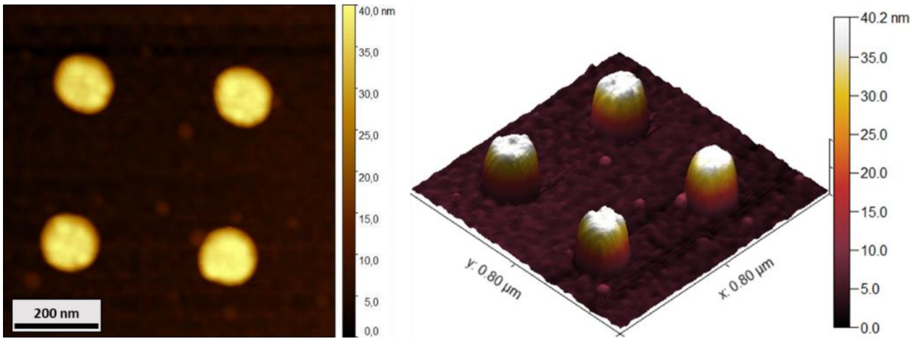

Figure S1. AFM pictures in 2D and 3D views of the gold nanodisks on gold film for D = 110 nm and P = 400 nm.

1.2. TERS measurements

The TERS colored map shown in Figure 3 in the main article represents a superposition of the Raman signal contributions associated with three areas of interest in the spectrum to different spectral ranges. The blue, green, and red maps from figure S.2 correspond respectively to the intervals 855-1272 cm^{-1}, 1375-1780 cm^{-1}, 2610-3215 cm^{-1} with characteristic peaks of the molecules. For each spectral range, the corners of the nanostructures show a higher signal in agreement with the higher electric field. Then, the final map in S2.c is obtained by superposition.

(a)

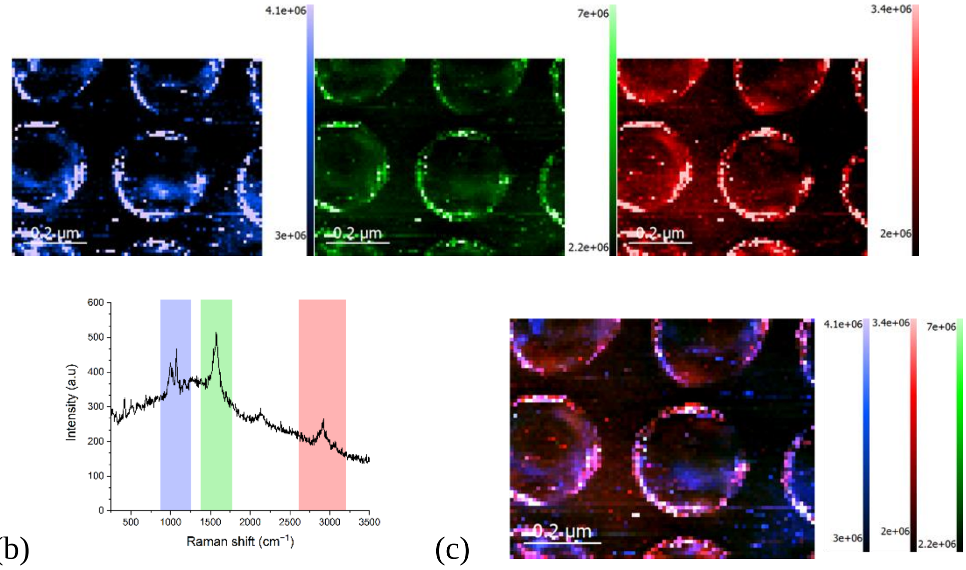

Figure S2. TERS mapping of 220 nm gold nanodisks with 638 nm laser. Each region of the Raman spectra can be associated with area on the sample (a-b). Then, we can superpose the area (c). In the TERS image, the pixel size is 10×10 nm^2.

In the same way, as for 220 nm gold nanodisks, we acquire the TERS image of the 110 nm gold nanodisks shown in figure S3 (a) with the corresponding Raman spectra (b) at the edge of the nanodisk (grey region & black curve), between the nanodisks (green region & curve) and at the top of the structure (red region & curve). The TERS image was obtained with a 638 nm, 80 µW lasers with a 60° incidence angle. The pixel size is 16 x 16 nm^2. As for the 200 nm gold nanodisks, the TERS signal is more intense at the edge of the nanodisks. The slight drift during the acquisition does not perturb the analysis. The Raman signal for the 1575 cm^{-1} peak is 1.8 ± 0.3 higher at the edge of nanodisks than at the top or between them.

(a)

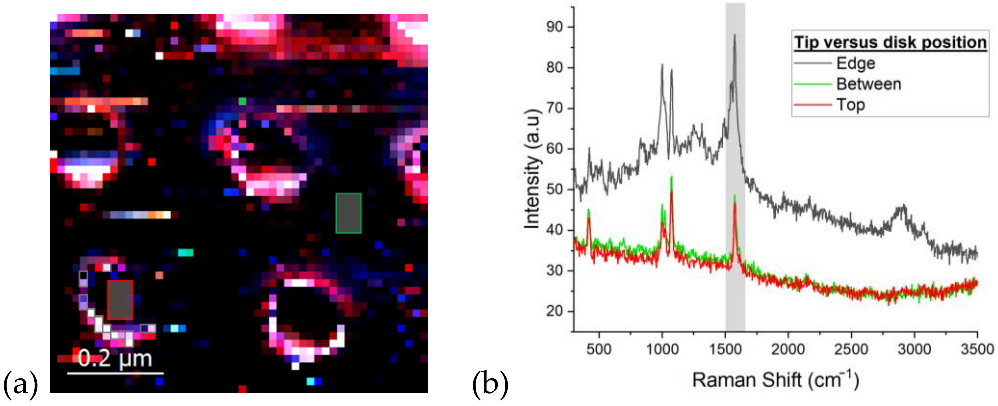

Figure S3. TERS mapping of 110 nm gold nanodisks with 638 nm laser. b) Raman signal for three identified sample regions: Top – Edge – Between.

1.3. Localized functionalization revelead by TERS

As described in the 1st and 2nd steps of Figure 8.a in the main article, we used a resist protective layer to functionalize only the gold nanostructures. Then, we performed TERS measurement at the end of the 2nd step to confirm there is no thiophenol molecules between the nanostructures. Several positions have been used and no characteristic Raman peak where observed between gold nanostructures (Figure S.4, green curve). On the contrary, we observed the thiophenol Raman signature when the measurement was done on

the top of the nanodisks (red curve), thus confirming our hypothesis of spatially-localized functionalization.

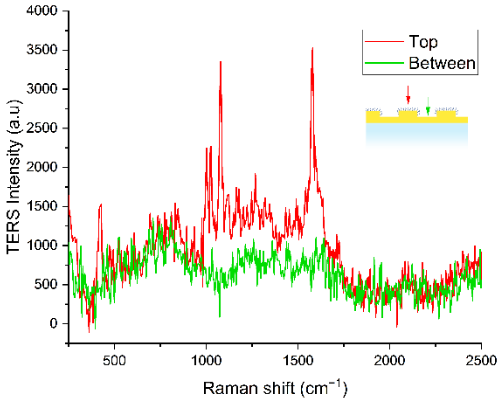

Figure S4. TERS intensities signal obtained from the top (red curve) and between (green curve) the nanostructures (220 nm gold nanodisks).

1.4. SERS measurements

A double functionalization experiment was performed on 100 nm disks (another set of samples) for three periods and three excitation wavelengths with a XploRA confocal Raman. Similar to the case of the 220 nm disks (Fig. 8.c), we obtain a ratio close to 1 between the two SERS measurements indicating that a major part of the signal comes from the nanostructure.

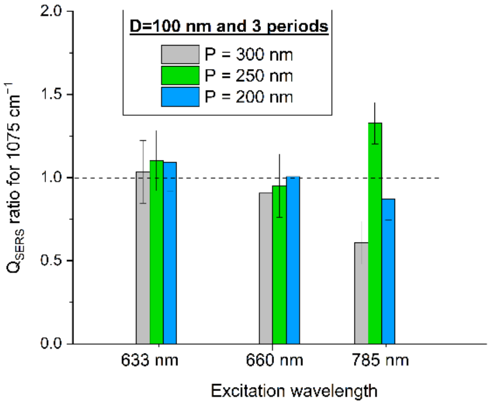

Figure S5. Ratio of SERS intensities between the first and second functionalization for three excitation wavelengths (633, 660, and 785 nm) for 1075 cm^{-1} characteristic peaks.

Finally, SERS measurements using a LabRAM Soleil Raman microscopy platform were also carried out on samples used in TERS to confirm the characteristic far-field peaks, suitable for non-laboratory measurements. Thus, we use three-excitation wavelengths available in the commercial setup (532, 638, and 785 nm). We obtain the characteristic peaks of thiophenol for the different wavelengths with a stronger signal for 638 nm and 785 nm. In figure S6, the number of metallic nanostructures under the laser beam is the same for both diameters, but the fill factor for 220 nm nanodisks is four times greater than for 110 nm. Thus, it would be expected that the intensity of the SERS signal would be more significant with the 220 nm nanodisks. However, the SERS spectra in fig S.6 are similar for the two nanodisk diameters. SERS intensity is related to the electric field amplification

and excitation wavelength. A higher electric field amplification could compensate for the lower fill factor of 110 nm diameter nanodisks for the excitation wavelength used. Also, experimental parameters such as the laser focus and the roughness may influence the SERS spectra intensity of 110 and 220 nm nanodisks diameter. Out of the nanostructures, no Raman peak signal is measured on the gold film.

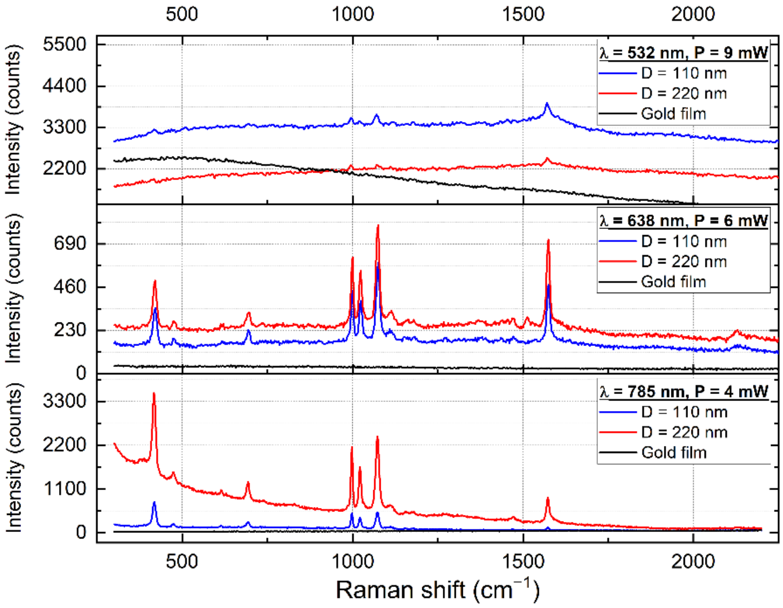

Figure S6. SERS spectra for two sizes of nanodisks (D = 110 and 220 nm) with a period of 400 nm. Three excitation wavelengths were used at 532, 638, and 785 nm.
